# On-Chip Electromembrane Surrounded Solid Phase Microextraction for Determination of Tricyclic Antidepressants from Biological Fluids Using Poly(3,4-ethylenedioxythiophene)—Graphene Oxide Nanocomposite as a Fiber Coating

**DOI:** 10.3390/bios13010139

**Published:** 2023-01-14

**Authors:** Razieh Zamani, Yadollah Yamini

**Affiliations:** Department of Chemistry, Faculty of Basic Sciences, Tarbiat Modares University, Tehran 14115-175, Iran

**Keywords:** electromembrane extraction, electromembrane surrounded solid phase microextraction, on-chip, microfluidics, tricyclic antidepressants, poly(3,4-ethylenedioxythiophene)—graphene oxide, biological fluids, human bone marrow aspirate

## Abstract

In the present study, on-chip electromembrane surrounded solid phase microextraction (EM-SPME) was employed in the determination of tricyclic antidepressants (TCAs), including amitriptyline, nortriptyline, imipramine, desipramine, maprotiline, and sertraline, from various biological fluids. In this regard, poly(3,4-ethylenedioxythiophene)–graphene oxide (PEDOT-GO) was electrodeposited on an SPME fiber as a conductive coating, then the fiber played the acceptor-electrode role during the extraction. Thus, the immigration of the analytes under the influence of an electric field and their absorption onto the fiber coating were accomplished simultaneously. Under the optimized conditions, the limits of detection for the target analytes were acquired in the range of 0.005–0.025 µg L^−1^ using gas chromatography–mass spectrometry. The linearity of the method was 0.010–500 µg L^−1^ for the imipramine and sertraline, 0.025–500 µg L^−1^ for the amitriptyline, nortriptyline, and desipramine, and 1.000–250 µg L^−1^ for the maprotiline (R^2^ ≥ 0.9984). Moreover, this method provided suitable precision and fiber-to-fiber reproducibility, with RSDs ≤ 8.4%. The applicability of the proposed setup was eventually investigated for extraction of the drugs from human bone marrow aspirate, urine, plasma, and well water samples, in which satisfactory relative recoveries, from 93–105%, were obtained.

## 1. Introduction

**Figure 1:**
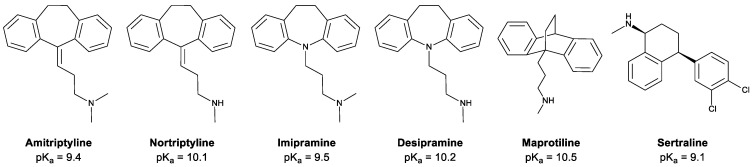
Chemical structures of the model TCAs.

Antidepressants are widely accepted as first-line medications for the treatment of major depressive disorder and other diseases such as clinical depression, anxiety [1], obsessive–compulsive disorder (OCD) [2,3], some chronic pains [4], persistent depressive disorder (dysthymia) [5], premenstrual syndrome (PMS) [6], preventive treatment of migraines [7], attention-deficit/hyperactivity disorder (ADHD) [8], post-traumatic stress disorder (PTSD) [9], and therapeutic management of some addictions and drug-seeking behaviors [10], in some cases. However, it has been affirmed that the consumption of antidepressants is commonly comorbid with some side effects, including discontinuation syndrome [11], nausea, constipation, dizziness, headaches, dry mouth followed by drowsiness, palpitations, and sweating [12], weight gain [13], increased risk of suicidal behavior [14], and sexual dysfunction [15]. Amitriptyline, nortriptyline, imipramine, desipramine, maprotiline, and sertraline are known as the most conventional class of tricyclic antidepressants (TCAs). Figure 1 illustrates the structure of these compounds.

Above and beyond all other considerations, the narrow therapeutic index (TI) of TCAs (50–300 μg L^−1^) necessitates monitoring concentrations of these drugs in human fluids in order to reduce the toxicity and side effects of the drugs, as well as pharmacokinetic studies, forensic pathology investigations, and postmortem examinations of bone marrow aspirate (BMA) [16]. Due to the problems of analysis of biological samples such as protein-based matrix interferences and low-concentration measurement, the sample preparation prior to analysis has been prescribed as the most fundamental step in a pharmaceutical analysis [17,18]. Therefore, numerous sample preparation methods with respect to capability, facilitation, simplification, and environmental sustainability sample preparation methods have been developed in the last few decades [19].

Electromembrane extraction (EME) was introduced by Pedersen-Bjergaard and Rasmussen for the first time in 2006 [20]. This sample preparation method is a miniaturized three-liquid-phase extraction (TLPE) technique developed for the extraction of ionized compounds from aqueous samples. A typical EME device comprises a donor phase for the sample solution, an acceptor phase, and a water-immiscible organic solvent, called supported liquid membrane (SLM), sustained in a porous hollow fiber membrane between the two phases. Thus, the sample solution and acceptor solution are not essentially contiguous and this merit leads to a sufficient sample clean-up strategy. The principle of the extraction procedure consists of the electrokinetic migration of the ionic analytes across the SLM into the acceptor phase solution under the influence of an electric potential. The high preconcentration, sensitivity, adequate precision, adaptability, simplicity, high clean-up, and µL-volume consumption of organic solvents make this technique an impressive and convincing green extraction method for analytical approaches [21].

Along with the recent developments of EME, electromembrane surrounded solid phase microextraction (EM-SPME), an electrically enhanced solid-phase technique, was reported by Yamini et al. in 2013 [22]. The EM-SPME system is defined by utilizing an SPME fiber in the acceptor phase, wrapped in the membrane in order to prevent contact with the sample solution. The combination of the EME and fiber SPME methods would undoubtedly exploit the positive features of both methods.

The matrix effect is the unintended interference or alteration in the results of an analytical measurement caused by non-target components of the sample. Memory effect is also the inaccuracy and non-repeatability in the results that originate from undesorbed analytes from the solid phase. Hence, it reduces the fiber lifetime to show a stable response. Since the acceptor phase is separated from the sample solution by the membrane in EM-SPME, the matrix effect and memory effect are minimized. Accordingly, EM-SPME can overcome the drawbacks of direct immersion SPME (DI-SPME), including the matrix effect and fiber lifetime, as well as the limitations of conventional EME, such as ultra-trace analysis and online coupling with chromatographic instruments [22]. Furthermore, the sensitivity of the method is enhanced dramatically due to the fact that all of the target analytes absorbed into the fiber coating can be introduced into the chromatographic column. Therefore, the main advantages of EM-SPME can be listed as remarkable efficiency and sensitivity, high clean-up, minimized organic solvent consumption, and low-concentration determination of ionizable compounds in complex sample matrices [23].

By means of this integrated method, extraction efficiency is claimed to increase in that the SPME fiber is employed as the acceptor-phase electrode, and the analytes would directly approach the coating after the migration across the SLM. Consequently, the SPME fiber should be a conductive material to be able to play the electrode role and apply the electric potential. Conductive polymers (CPs) are recognized as one the most appropriate materials to conduct electricity with satisfactory electrical properties. The simplicity of the chemical and electrochemical synthesis of CPs in aqueous and non-aqueous solutions, and the diversity in pendant groups with different features, could be suggested as the most desirable characteristics of conductive polymers. Poly(3,4-ethylenedioxythiophene) (PEDOT) could be mentioned as a highly conductive polymer (~300 S cm^−1^ varies with the dopant type and polymerization process) with superior chemical and thermal stability, as well as environmentally friendly properties [24,25].

In addition to the approach to achieving the most favorable qualities in the analytical methods; automating the process, accelerating measurement, reducing energy and reagents consumption, and price regulation have been the main objectives for analytical studies over the past two decades. Thus, the micro-total-analysis system (µ-TAS) was proposed with the intention of facilitating experiments. In recent years, µ-TAS has been growing exponentially and has evolved into the most common scientific and industrial research areas [26]. Microfluidic devices, also known as lab-on-chip (LOC), have acquired the design of an integrated and miniaturized system capable of performing a series of functions automatically [27,28]. The downscaling of EME devices on a chip platform was executed by Petersen et al. in 2010 for the first time [29]. Hence, on-chip EME would assuredly benefit from the positive aspects of microfabricated systems mentioned before [30].

Herein, an on-chip EM-SPME device was proposed for the first time, and it was employed for the determination of tricyclic antidepressants in various biological fluids as a solvent-free sample preparation method. Therefore, a poly(3,4-ethylenedioxythiophene)—graphene oxide (PEDOT-GO) nanocomposite was electrochemically synthesized on an SPME playing the role of the negative electrode during the extraction. The function of the proposed system was evaluated for the determination of amitriptyline (Ami), nortriptyline (Nor), imipramine (Imi), desipramine (Des), maprotiline (Map), and sertraline (Ser) using gas chromatography–mass spectrometry (GC–MS) in bone marrow aspirate, urine, plasma, and well water samples.

## 2. Experimental

### 2.1. Chemicals and Materials

Amitriptyline, nortriptyline, imipramine, desipramine, maprotiline, and sertraline were supplied by Razi Distribution Co. (Tehran, Iran). The stock solutions of the antidepressants were prepared in methanol (100 mg L^−1^) and stored at 4 °C. Lower concentrations were prepared daily by diluting the stocks with HCl solution to adjust the desired pH. Sulfuric acid (H_2_SO_4_) and 2-nitrophenyl octyl ether (NPOE) were purchased from Fluka (Buchs, Switzerland). Potassium persulfate (K_2_S_2_O_8_), phosphorus pentoxide (P_2_O_5_), potassium permanganate (KMnO_4_), sodium perchlorate (NaClO_4_), and 3,4-ethylenedioxythiophene (EDOT) were obtained from Sigma-Aldrich (Milwaukee, WI, USA) for the synthesis of the nanocomposite. Graphite powder (325 mesh) was supplied by Neutrino (Tehran, Iran). Acetonitrile was also purchased from Sigma-Aldrich. Hydrogen peroxide (H_2_O_2_), sodium chloride (NaCl), and hydrochloric acid (HCl) were obtained from Merck (Darmstadt, Germany). All of the used chemicals and reagents in this work were of analytical grade. For the fabrication of the chip, 1.0 and 3.0 mm thick poly(methyl methacrylate) (PMMA) plates were supplied by Royal Plast (Tehran, Iran). The 316 Stainless steel electrodes (d = 0.25 mm) were purchased from Ghaem Metal Industrial (Tehran, Iran). The synthesis of the nanocomposite coating was carried out on a spinal needle with a gauge number of 27 and a length of 2.5 cm. The flat sheet polypropylene membrane, with a thickness of 100 μm, 55% porosity, and pore size of 0.2 μm, was obtained from Membrana (Wuppertal, Germany). The nylon net filter (0.2 μm) and cellulose acetate membrane filters (0.45 µm pore size) were purchased from MF-Millipore (Madrid, Spain).

### 2.2. Instruments and Apparatus

The microfluidic chip fabrication and cutting of the PMMA plates with the required patterns were accomplished using a laser cutting machine from Perfect Laser Co., Ltd. (Wuhan, China). A Tecno-Gaz S.p.A. ultrasonic bath model 2092/U (Parma, Italy), a Heidolph MR 3001K hotplate with a magnetic stirrer (Kelheim, Germany), and a Zhaoxin RXN-305D power supply (Zhaoxin, China) were employed during the synthesis of the nanocomposite. For the real sample preparation, a Selecta Lab centrifuge, model TL320, (Miami, FL, USA) was used. The used ultrapure water was also produced by a Young Lin aquaMax Ultra 370 Series water purification system (Kyounggi-do, Republic of Korea). The morphology of the synthesized nanocomposite was investigated using a TESCAN field-emission scanning electron microscope (FE-SEM) model MIRA3 XMU (Brno, Czech Republic). For indicating the formation of the intended nanocomposite, a Thermo Nicolet Fourier-transform infrared spectrometer (FT-IR) model IR100 was employed (Madison, WI, USA). To determine the thermal stability of the synthesized coating, a TG 209 F3 Tarsus thermogravimetric analyzer from NETZSCH was employed (Bavaria, Germany). Moreover, a zeta potential analyzer, model Zetasizer Nano ZS, from Malvern Panalytical (Worcestershire, UK) was used in order to find out the point of zero charge (PZC) of the nanocomposite. The pumping of the sample solution into the donor chamber was carried out with a syringe pump from Fanavaran Nano-Meghyas (Tehran, Iran). During the extraction procedure, the required electric potential was applied between the positive electrode and the SPME holder piercing needle using a Paya Pajoohesh Pars power supply model 8760 T3 (Tehran, Iran) with adjustable output voltage and current in the range of 0–600 V and 0–500 mA, respectively. A 25 µL Hamilton syringe (model 702 N) was employed for the impregnation of the polypropylene membrane with SLM (Bonaduz, Switzerland).

The optimization of the effective parameters on the extraction and conditioning of the fibers was performed on an Agilent 7890A gas chromatograph system (GC) equipped with a flame ionization detector (FID) and split/splitless injection port (Palo Alto, CA, USA). An Agilent CP-Sil 8 CB GC column (30 m × 0.32 mm × 0.25 μm) was employed for the separation of the analytes. The flow rate of helium (99.999%) as the carrier gas was set at 2 mL min^−1^. Moreover, the injector and detector temperatures were adjusted to 300 °C. For the quantitative survey and real sample analysis, an Agilent 7890B GC-5977B mass spectrometric detector (MS) with a split/splitless injection port was employed, and the MS was operated in the electron impact ionization mode (70 eV). An Agilent HP-5 GC column (30 m × 0.25 mm × 0.25 μm) was used for the separation of the antidepressants. The flow rate of helium (99.999%) as the carrier gas was adjusted to 1 mL min^−1^. The ion source and interface temperatures were both set to 300 °C. The separation of the TCAs on the GC–MS was achieved using the following temperature program: The initial temperature of the column was set at 45 °C and held for 2 min, increased to 290 °C at a rate of 15 °C min^−1^, then held for 2 min to remove the residual contaminants in the column. The SPME holder was introduced to the injector in the splitless mode while the split valve was kept closed for 5 min for all the experiments. Moreover, to achieve the highest sensitivity for each analyte, the MS analyzer was operated at a time-scheduled selected ion monitoring mode (SIM) (Table S1).

### 2.3. Fabrication of EM-SPME Chip Device

**Figure 2:**
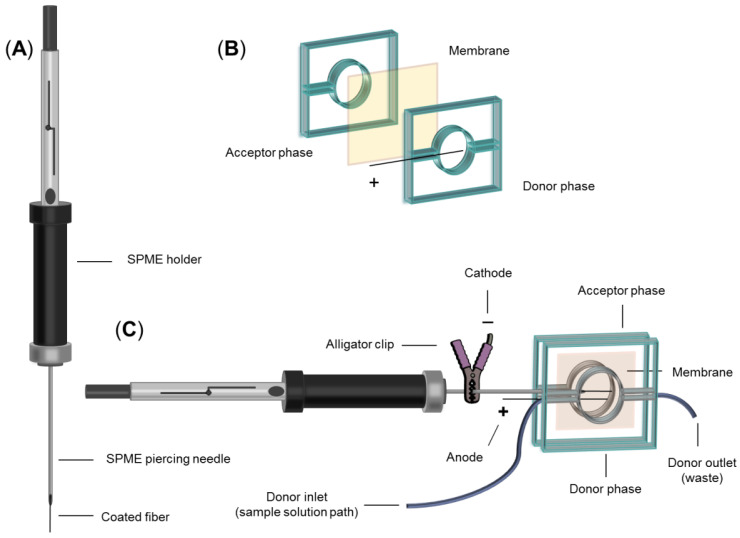
(**A**) Schematic of the SPME holder containing coated fiber; (**B**) schematic of the employed microfluidic device; and (**C**) assembled setup for on-chip EM-SPME.

The schematic diagram of the designed chip pattern employed for the EM-SPME technique is illustrated in Figure 2. The chip comprises two 35 mm × 35 mm PMMA plates wherein a circular chamber, with a diameter of 15 mm and a depth of 1.0 mm, was embedded as the donor and acceptor chambers. Accordingly, an inlet was added for the insertion of the SPME piercing needle into the acceptor phase, in addition to the inlet and outlet routes for the solutions (i.d. of 0.7 mm). Another hole was also drilled to install the stainless steel electrode into the donor phase chamber. Prior to each extraction, a piece of 20 mm× 20 mm polypropylene membrane was impregnated with the SLM and situated between the two phases. Ultimately, the chip was completely sealed with the aid of two clamps compressing the phases together.

### 2.4. Synthesis of PEDOT-GO Nanocomposite

Initially, graphene oxide (GO) was synthesized according to a modified Hummers method [31]. In this way, 3 g of natural graphite powder (325 mesh) was added to 12 mL of concentrated sulfuric acid containing 2.5 g of K_2_S_2_O_8_ and 2.5 g of P_2_O_5_ at 80 °C and stirred for 4.5 h. This mixture was diluted with 0.5 L of ultra-pure water after cooling down to room temperature. The diluted mixture was filtered using a nylon net filter and washed with ultra-pure water to remove the residual H_2_SO_4_. Then, the obtained powder was dried under ambient lab conditions. This pre-oxidized graphite powder was again added to 120 mL of concentrated H_2_SO_4_ at 0 °C. Successively, 15 g of KMnO_4_ was gradually added while stirring for 2 h, and the temperature was kept below 35 °C. Then, 250 mL of ultra-pure water was added to the mixture in an ice bath and stirred for 2 h. Afterward, another 0.7 L of ultra-pure water and 20 mL of H_2_O_2_ (30%) were added to the mixture, at which time the color changed to bright yellow. The obtained yellowish mixture was filtered and washed with 1 L of HCl solution (1:10) followed by 1.0 L of ultra-pure water to remove the residual metal ions and acid, respectively. Ultimately, this resulting GO powder was dried overnight.

The PEDOT-GO nanocomposite was synthesized on the SPME fiber by an electrodeposition technique [32]. Before starting the electropolymerization process, the stainless steel fiber was immersed into the HCl solution (30%) for chemical etching for 30 min, and then was washed with distilled water, acetone, methanol, and acetonitrile, respectively. Then, 20 mg of the GO, as the anion dopant for polymerization, was well dispersed in ACN solution containing an EDOT monomer (0.1 M) to obtain a 1 mg mL^−1^ mixture of GO. After 60 min of sonication, two 1.5 cm length stainless steel fibers, employed as the electrodes, were situated in the homogenous mixture. The electrodeposition process was carried out by applying a constant potential of 3.0 V for 30 min. The structure and mechanism of the electrochemical deposition of PEDOT-GO are illustrated in Figure S1 (Supplementary Materials). After the polymerization, the black coating, formed on the anode electrode, was washed with ultra-pure water and eventually dried at room temperature for further experiments. In order to prepare the fiber and remove possible contamination, the fiber was inserted into the GC injection port to be conditioned at 220 °C for 1 h. To provide more reassurance, the temperature program used for the separation of analytes was implemented on it afterward. The synthesis of the PEDOT polymer coating was performed by repeating the mentioned steps, where NaClO_4_ (0.2 M) was employed as the anion dopant and a voltage of 2.6 V was applied between the electrodes.

### 2.5. Extraction Procedure

A 20 mm × 20 mm piece of polypropylene membrane was impregnated with 40 μL of NPOE using a Hamilton syringe and situated between the donor and acceptor phases. The chip was completely sealed with two clamps compressing the phases together. Then the acceptor phase was filled with a sufficient amount of solution to overflow. The piercing needle of the SPME holder was inserted into the acceptor phase chamber, and the fiber was exposed to the solution. The upper part of the piercing needle was connected to the negative pole of a power supply to represent the cathode in the acceptor phase, whereas the donor phase electrode was connected to the positive pole (anode). The extraction procedure was started by pumping 2 mL of the sample solution into the donor chamber with a syringe pump, along with applying the required voltage. After the accomplishment of the immigration of the target analytes across the membrane, and also the adsorption of them onto the nanocomposite, the fiber was retracted back into the holder. The SPME holder was then taken out and introduced to the GC injection port. In this stage, the analytes on the fiber coating were thermally desorbed in the injector and directly transferred to the GC column for separation. Eventually, the chip chambers were washed with ultra-pure water and the disposable membrane was renewed for the subsequent extractions.

### 2.6. Real Sample Preparation

Urine specimens from a participant who consumed a 50 mg imipramine tablet were collected right before the consumption (labeled as 0 h) and at 1, 2, 4, 6, 9, 12, and 24 h after the consumption, then stored at 4 °C. For further experiments, a dilution of 100 times was performed using ultra-pure water. A drug-free plasma sample acquired from healthy donors from the Iranian Blood Transfusion Organization (Tehran, Iran) was obtained and stored at −18 °C. The plasma sample was also diluted 1:20 before the analysis. A bone marrow aspirate sample of a test subject, diagnosed with cancer, who had taken a 50 mg sertraline tablet every two weeks was obtained by an oncologist in the pediatric stem cell transplantation ward of Children’s Medical Center (Tehran, Iran). The BMA sample was collected in a pink-top tube (containing EDTA as an anticoagulant) and stored at −18 °C before use. In order to separate the blood stem cells and platelets from the sample, the BMA was centrifuged at 5000× *g* rpm for 5 min. Further, the stage of fat removal was performed. Accordingly, 150 µL of 1% formic acid solution and 800 µL of ethanol were added to 3 mL of the BMA sample, and then the sample was vortexed for 5 min. As a consequence, the fat was transferred to the non-polar phase. Afterward, the sample was gently shaken for 10 min, and then centrifuged at 10,000× *g* rpm for 10 min to separate the fat cells. Ultimately, the lower phase was separated, purged with nitrogen in order to evaporate the organic solvent, and diluted 10 times. All the biological samples were diluted to keep the detected results of the experiments within the linear range, and the mentioned dilution rates were obtained from experience. A well water sample was also supplied from a private well of Tarbiat Modares University (Tehran, Iran). To prepare all the real samples prior to the extraction procedure, the pH of the samples was adjusted to 4 (the optimal donor pH), and then the sample solutions were filtered using a cellulose acetate syringe filter.

## 3. Results and Discussion

The anodic oxidation of the EDOT monomers on the fiber surface begins with the formation of cation radicals and their coupling for progressive growth. The GO sheets possess numerous anionic groups, such as carboxylic acid (−COOH), hydroxide (–OH), and epoxide (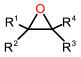). Thus, GO dopant with a negative charge could be incorporated during the reaction into the PEDOT structure to maintain electrical neutrality. Subsequently, the nanocomposite would be electrodeposited on the fiber as a result of electrostatic interaction [33].

### 3.1. Characterization of PEDOT-GO

**Figure 3:**
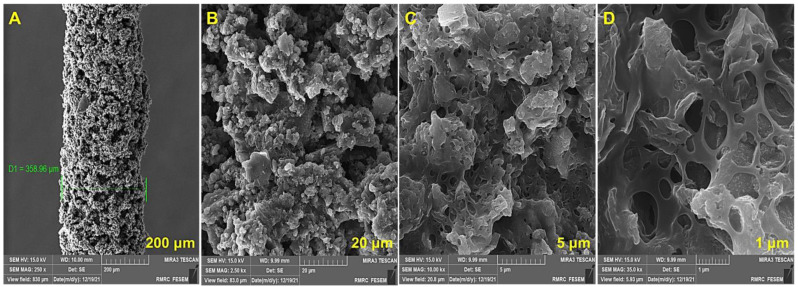
FE-SEM images of PEDOT-GO nanocomposite.

The surface morphology of the fiber coating was characterized by FE-SEM (Figure 3). The forming of a uniform 44-micron thick coating on the fiber surface is demonstrated, and no sign of accumulation on the GO sheets is seen. These clear illustrations confirm the highly porous and 3-D crumpled structure of the nanocomposite [34]. As is corroborated, the porosity of the PEDOT-GO structure and its interconnected network of holes indicate the existence of many active sites for interaction with the desired compounds, which would lead to the enhancement of extraction efficiency.

**Figure 4:**
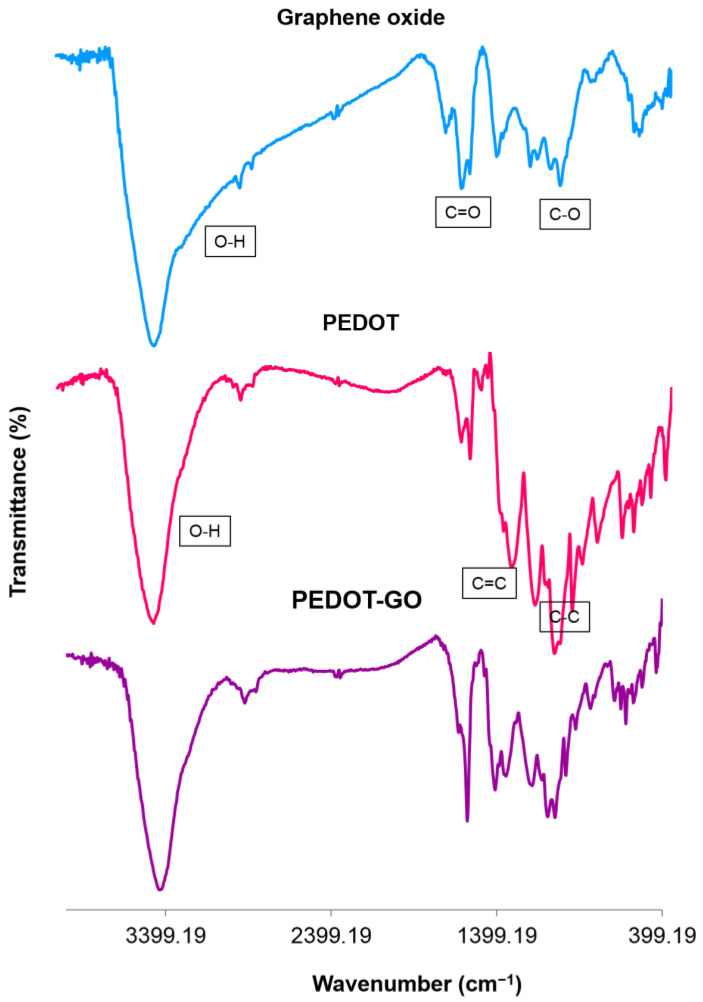
FT–IR spectra of GO, PEDOT, and PEDOT–GO nanocomposite.

The chemical structures of the PEDOT-GO nanocomposite, PEDOT and GO, were assessed by FT-IR spectroscopy (Figure 4). The GO powder spectrum contains characteristic absorption bands at 3400, 1700, and 1039 cm^−1^ referred to O–H, C=O and C–O stretching vibrations, respectively [32]. In the spectrum of the PEDOT polymer, a strong absorption band at 3400 cm^−1^ related to the O–H stretching vibration, and 1575 and 1317 cm^−1^ are assigned to C=C asymmetric stretching and C–C stretching in the thiophene heterocycle, respectively. The observed absorption peaks around 1184, 1072, and 1047 cm^−1^ are attributed to the C–O–C stretching vibrations in the ethylenedioxy group. In addition, the absorption bands at 975, 916, 825, and 684 cm^−1^ were due to the stretching vibration of the C–S–C bond in thiophene [35]. The spectrum of PEDOT-GO indicates a combination of GO and PEDOT spectra, which confirms the synthesis of the desired nanocomposite. The absorption peaks at 2892 and 2937 cm^−1^, which referred to the stretching vibration of –CH_2_ in the thiophene heterocycle, shifted to a higher position, by about 27–44 cm^−1^, compared to the PEDOT (blue shift). This shift signified that there is a specific interfacial interaction between the PEDOT molecular chains and the GO. Therefore, these interactions would stabilize the nanocomposite, which leads to witnessing an increase in the required energy for vibration and thus wavenumber. All these indicators confirm the successful formation of PEDOT-GO in the polymerization reaction.

Inasmuch as the fiber was involved in the thermal desorption stage, the synthesized coating had to be of high endurance and durability. Therefore, a thermogravimetric analysis (TGA) was employed to estimate the thermal stability of the nanocomposite, and the result is illustrated in Figure S2. This analysis was recorded under a nitrogen atmosphere from room temperature increasing to 600 °C, with a heating rate of 10 °C min^−1^. A gradual mass loss was observed in the temperature range of 290–470 °C, attributed to the decomposition of carboxylic acid and other oxygenated functionalities. The mass loss was then followed by a slight continuous decrease up to 600 °C, which could have occurred due to the elimination of the residual polymer components. The TGA indicated that PEDOT-GO is a sufficient nanocomposite to be used as a fiber coating in this work [35].

The zeta potential (ZP), also known as the electrokinetic potential in a colloidal system, is a function of the particles’ environment, such as pH. Thus, a zeta potential analysis could be employed to investigate the surface charge of the fiber coating at different pH values. In this way, a colloidal dispersion of PEDOT-GO (0.5 mg mL^−1^) was examined over a pH range of 2.5–7 at 25 °C. As shown in Figure S3, the pH_pzc_ (point of zero charge) of the nanocomposite is approximately 3.8, which indicates that the net charge of the total PEDOT-GO surface is zero. Accordingly, the surface charge is negative at pH > pH_pzc_ due to the deprotonation of functional groups, whereas it becomes positively charged at pH < pH_pzc_ [36,37]. The rise in the ZP magnitude, along with the increase in pH values, is a result of an increase in the surface charge density on the PEDOT-GO particles. Considering all the above, it is evident that the acceptor phase pH exerts a significant influence on antidepressants’ adsorption on the fiber coating.

### 3.2. Optimization of Effective Parameters on the Extraction

**Figure 5:**
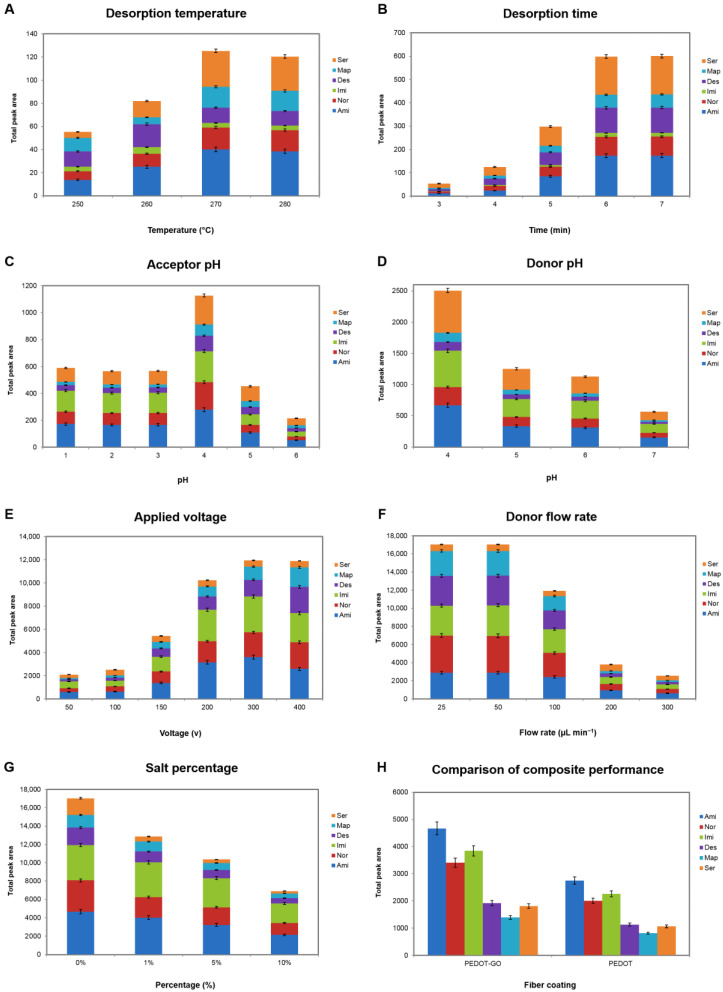
(**A**–**G**) Optimization of effective parameters on extraction procedure. Extraction conditions for the first optimization experiments (desorption temperature) include: desorption time: 4 min, acceptor pH: 1, donor pH: 6, voltage: 100 V, sample solution flow rate: 200 µL min^−1^, and in the absence of salt; and (**H**) comparison of the capability of the PEDOT-GO and PEDOT fiber coating in the extraction of TCAs via proposed chip. Extraction was carried out under the optimized conditions: desorption temperature: 270 °C, desorption time: 6 min, acceptor pH: 4, donor pH: 4, voltage: 300 V, sample solution flow rate: 50 µL min^−1^, and in the absence of salt.

The optimization of the effective parameters on the extraction and desorption of antidepressants was carried out using a monothetic analysis (one-variable-at-a-time) with the aim of achieving the highest efficiency. These parameters were prioritized as desorption temperature, desorption time, acceptor pH, donor pH, applied potential, sample solution flow rate, and salt effect, respectively (Figure 5). In all of the chromatograms, the areas related to the peak of each analyte are summed and reported. Thus, the changes in the total peak area values are directly related to the changes in the extraction efficiencies of all the analytes.

#### 3.2.1. Desorption Temperature

To ensure the immediate and efficient transfer of the compounds of interest from the fiber coating into the GC column, as well as maintaining the stability of the fiber, the inlet temperature should be optimized. Furthermore, the increasing temperature leads to the minimization of carryover effects. As is depicted in Figure 5A, the extraction efficiencies showed an upward trend by increasing the desorption temperature to 270 °C and remained approximately constant afterward. Therefore, in order to extend the lifetime of the fiber, 270 °C was selected as the optimized temperature for the desorption stage.

#### 3.2.2. Desorption Time

It is essential for the fiber to remain in the injection port for a sufficient time so that the analytes have the opportunity to transfer to the column. In this way, the desorption time was investigated in the range of 3–7 min. As seen in Figure 5B, extraction efficiencies experienced a sharp rise from 3–6 min. Thus, 6 min was employed as the desorption time for further experiments.

#### 3.2.3. Acceptor pH

According to the Nernst–Planck equation simulated by Pedersen-Bjergard et al., there is an inverse relationship between the flux of the analytes across the SLM and the ion balance (χ) in the EME system [38]. The ion balance represents the concentration ratio of the total ions in the acceptor phase to the total ions in the donor phase. This equation demonstrates that the higher ionic concentration of the acceptor solution (relative to the donor phase) inclines the analytes to immigrate to the acceptor solution. Consequently, the pH of the acceptor solution is a vital parameter during the extraction. Due to the electrolysis process of water, the pH of the acceptor phase gradually increases during the extraction. In addition, the acceptor solution should be completely acidic to prevent the back extraction of the analytes into the donor phase. To investigate the acceptor pH influence on extraction, the pH was changed from 1–6 using a 0.1 M HCl solution (Figure 5C). The efficiencies increase along with the decrease in pH from 6 to 4, due to the decline in the ion balance, then dropped from 4 to 3 and witnessed a steady trend to pH = 1. Thus, the highest extraction efficiencies were attained at pH = 4. This result is consistent with the obtained pH_pzc_ for the PEDOT-GO nanocomposite. By way of illustration, since the surface charge of the coating is negative at pH > 3.8, the fiber coating has no tendency to absorb cationic compounds. Thus, the absorption of antidepressants onto the SPME fiber decreased at pH < 3.8, and pH = 4 was eventually selected as the optimal acceptor pH.

#### 3.2.4. Donor pH

EME is an operative technique for the extraction of ionic compounds under the influence of an electric potential. Therefore, as the weakly basic drugs are required to be in their cationic form, the antidepressants’ sample solution must be strongly acidic. At pH ≤ pK_a_ − 2, approximately 100% of the analytes are in their ionized form. In order to investigate the effect of the donor pH on extraction, the pH of the sample solution was changed in the range of 4–7. According to Figure 5D, the results indicate that the best extraction efficiencies were obtained at pH = 4. As the pK_a_ values for the antidepressants are enumerated in Figure 1, all of them are greater than 9.1. Therefore, the target analytes completely convert into their cationic form at pH = 4. Also, another interpretation of this trend could be the decrease in the ion balance caused by the decline in the donor pH, in accordance with the theory.

#### 3.2.5. Applied Voltage

Electromembrane extraction is directly dependent on the applied potential between both phases’ electrodes [38]. Accordingly, the applied voltage effect was inspected from 50–400 V (Figure 5E). The results signified that the extraction efficiencies of the antidepressants drastically increased from 50 to 300 V, and then remained constant. Therefore, the applied potential of 300 V was employed for the rest of the experiments.

#### 3.2.6. Sample Solution Flow Rate

Subsequently, the flow rate of the sample solution passing through the donor phase was optimized in the range of 25–300 µL min^−1^. Prolonging the extraction process leads to an increase in the extraction of the analytes by providing them enough opportunity to immigrate over the membrane (prior to the enlargement of the boundary layer with ions and disinclination of mass transfer). Conforming to the obtained result in Figure 5F, the extraction efficiencies rose significantly along with decreasing the flow rate of the sample solution to 50 µL min^−1^.

#### 3.2.7. Salt Percentage

As mentioned previously, the existence of salt in the sample solution increases the χ value and decreases the extraction efficiency, consequently. Moreover, the analytes of interest are obliged to compete with other cations in the sample solution in this situation. The salt influence on the extraction was investigated for up to 10% of the NaCl to avoid the production of a large electric current through the membrane. As depicted in Figure 5G, the antidepressants’ extraction efficiencies experienced a sharp drop by adding NaCl, in accordance with the expectations.

### 3.3. Method Validation

In order to compare the capability of the PEDOT-GO nanocomposite with the PEDOT for the adsorption of antidepressants, extractions with these two fibers were carried out under identical conditions. The obtained result in Figure 5H indicates that the PEDOT-GO fiber coating shows considerably better performance in the extraction of TCAs from an aqueous sample solution spiked with 250 µg L^−1^ of the TCAs. This distinction could be due to the high porosity of the nanocomposite, numerous active sites, and effective functional groups in the structure of PEDOT-GO in comparison with PEDOT pure polymer.

The validation of the on-chip EM-SPME procedure was performed under the optimized conditions to confirm the suitability of the method for the determination of TCAs. Table 1 itemizes the achieved figures of merit for each antidepressant. The limits of detection (LODs) of the target drugs were in the range of 0.005–0.025 µg L^−1^. The obtained linearity was 0.010–500 µg L^−1^ for Imi and Ser, 0.025–500 µg L^−1^ for Ami, Nor, and Des, and 1.000–250 µg L^−1^ for Map, with R^2^ ≥ 0.9984. The LODs were obtained based on reaching the S/N ratio equal to 3 (experimentally) to prove the presence of the analytes in the sample with a 99% probability. In addition, the proposed setup provided appropriate precision and reproducibility corresponding to the single fiber (inter-day and intra-day), and fiber-to-fiber relative standard deviations (RSDs) between 3.2 and 8.4% (*n* = 3).

The quantitative performance of this developed method was compared with some of the previous reports in Table 2. As demonstrated, appropriate LODs and comparable linear dynamic ranges (LDRs) to other scientific papers have been achieved.

### 3.4. Real Samples Analysis

**Figure 6:**
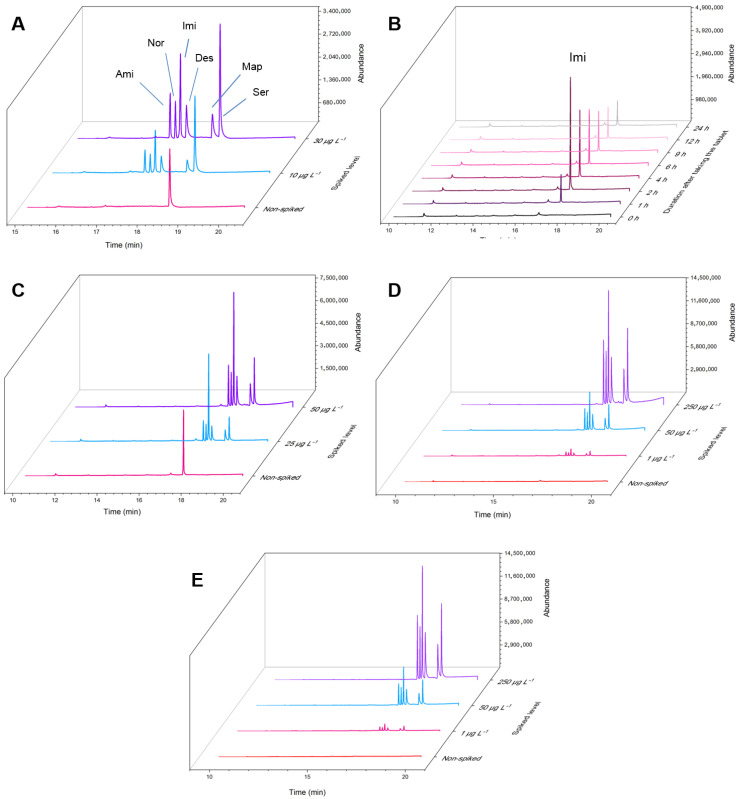
Obtained GC–MS chromatograms related to extraction of TCAs from (**A**) human bone marrow aspirate sample; (**B**) urine samples taken at different hours after consumption of imipramine; (**C**) urine sample taken 2 h after consumption of imipramine; (**D**) drug-free plasma sample; (**E**) well water sample, and their different spiked levels.

Electromembrane extraction was introduced as a powerful sample preparation method for the extraction of ionizable analytes from complicated real samples. To verify the applicability of the proposed EM-SPME device, the chip was employed for the extraction of the model analytes from human bone marrow aspirate, urine, plasma, and well water samples (Table 3, Table 4, Table 5 and Table 6, Figure 6). Considering the degree of dilution for the biological samples, there was no need to acquire the linearity of the TCAs in the matrices of the real samples.

As shown in Table 3, sertraline at a concentration of 32.0 µg L^−1^ was found in the BMA sample. To investigate the matrix effect on the accuracy and validity of the method, the sample was spiked with two concentration levels of 10.0 and 30.0 µg L^−1^ of antidepressants’ standard solution, and satisfactory relative recoveries (RRs) were achieved (>92%). Figure 6A illustrates the relevant overlaid chromatograms. The urine sample assessment at different times in a 24-h schedule was also depicted in Figure 6B. The correlated concentration-time curve for imipramine is plotted in Figure S4. The maximum concentration of 47.2 µg L^−1^ was found in the sample taken 2 h after consumption and was spiked with two concentrations of 25.0 and 50.0 µg L^−1^ (Figure 6C). None of the TCAs were found in the drug-free plasma sample. Thus, three different concentrations, of 1.0, 50.0, and 250.0 µg L^−1^ from the beginning, middle, and end of the linear range, were spiked. Figure 6D delineates the relevant 3-D chromatograms of this analysis. The obtained results in Table 6 show that the well water was free from antidepressant contamination. Therefore, this sample was also spiked with 1.0, 50.0, and 250.0 µg L^−1^ of the target TCAs, and proper relative recoveries, from 93–105%, were attained (Figure 6E).

## 4. Conclusions

This work, for the first time, has presented EM-SPME using a simple and inexpensive chip device in combination with a common DC constant power supply. The aim of this paper is to introduce the benefits of this new concept of an electrically enhanced extraction chip. It was anticipated that the introduced EM-SPME could increase the stability of the extraction system by fixing the electrode at defined positions and could increase the effective potential between the electrodes by decreasing their distance from each other. Thus, using this chip, the extraction efficiency of the analytes will be increased and the extraction time can be decreased in comparison with conventional EM-SPME.

An on-chip EM-SPME technique using a PEDOT-GO fiber as the acceptor electrode was employed for the extraction of tricyclic antidepressants from body fluids and well water, followed by GC–MS. The aim of this microfluidic device was to overcome the drawbacks of conventional EME and DI-SPME methods. As has been demonstrated, the presented device provided the analysis with high clean-up, low limits of detection, wide linearity, and appropriate extraction efficiencies. Assuredly, the minimization of organic solvent consumption as much as possible due to the solvent-free nature of SPME, in addition to the capability of coupling EME with gas chromatography, made this method a felicitous sample preparation method for the trend analytical approaches. Furthermore, the PEDOT-GO nanocomposite was recognized as a suitable fiber coating with high porosity, strong conductivity, proper thermal stability, and uniform decomposition. Numerous functional groups and active sites, and the ability to become negatively charged at specific pH, lead to the enhancement of extraction efficiencies. Likewise, the results of successive extractions showed that the fiber presented a lifetime of 100 extraction/desorption cycles without any significant change in its performance. A careful inspection of the nanocomposite structure indicated that electrostatic and π-π interactions, and also hydrogen bonding, are the involved mechanisms in the absorption of the antidepressants by the PEDOT-GO fiber. In conclusion, the on-chip EM-SPME possessed significant accomplishments in the determination of the target analytes in biological fluids and wastewater with adequate relative recoveries.

## Figures and Tables

**Table 1:** Figures of merit for proposed on-chip EM-SPME-GC–MS for TCAs from aquatic samples using PEDOT-GO nanocomposite.

Analyte	LOD (µg L^−1^)	LDR (µg L^−1^)	R^2^	Inter-Day RSD (%, *n* = 3)	Intra-Day RSD (%, *n* = 3)	Fiber-to-Fiber RSD (%, *n* = 3)
Amitrptyline	0.010	0.025–500	0.9989	4.5	5.3	7.2
Nortriptyline	0.010	0.025–500	0.9987	3.2	4.9	6.8
Imipramine	0.005	0.010–500	0.9996	4.3	2.9	5.3
Desipramine	0.010	0.025–1000	0.9986	5.1	5.4	6.1
Maprotiline	0.025	1.000–250	0.9984	4.8	6.2	8.4
Sertraline	0.005	0.010–500	0.9991	5.4	5.7	7.6

**Table 2:** Comparison of the proposed method with other relevant methods for determination of TCAs.

Analytical Method	Analytes	LOD (µg L^−1^)	LDR (µg L^−1^)	RSD (%)	Ref.
HF-LPME-GC–MS ^1^	8 TCAs (Ami, Nor, Imi, Des)	10.0	20.0–120.0	0.6–9.7	[39]
SPME-HPLC–UV ^2^	Ami, Chl ^3^	3.13–4.19	10–500	≤6.85	[40]
DLLME-GC–MS ^4^	5 TCAs (Ami, Nor, Imi, Des)	0.5–2.0	2.0–100	2.0–9.9	[41]
EME-GC–FID	Imi, Clo ^5^	0.35–0.70	2–1500	8.0–8.5	[17]
EC-IT-SPME-HPLC-UV ^6^	Ami, Nor, Imi, Dex ^7^	0.3–0.5	0.70–200	≤8	[42]
BAμE-GC–MS ^8^	6 TCAs (Am, Imi)	0.2–1.6	10.0–1000	≤13.9	[43]
MEPS-GC–MS ^9^	Ami, Imi	0.03–0.05	0.1–500	≤9.0	[44]
Mμ-SPE-GC–MS ^10^	Ami, Chl	0.008–0.010	0.05–500	≤7.9	[45]
On-chip EM-SPME-GC–MS	Ami, Nor, Imi, Des, Map, Ser	0.01–0.25	0.5–500	4.5–8.1	This work

^1^ HF-LPME: hollow-fiber liquid-phase microextraction; ^2^ HPLC-UV: high-performance liquid chromatography—UV; ^3^ Chl: chlorpromazine; ^4^ DLLME: dispersive liquid-liquid microextraction; ^5^ Clo: clomipramine; ^6^ EC-IT-SPME: electrochemically controlled-in tube SPME; ^7^ Dex: dexopin; ^8^ BAμE: bar adsorptive microextraction; ^9^ MEPS: microextraction in packed syringe; ^10^ Mμ-SPE: magnetic micro-solid phase extraction.

**Table 3:** Obtained results from the determination of TCAs in human bone marrow aspirate sample spiked at two different concentration levels.

Analyte	C_real_ (µg L^−1^)	C_add_ (µg L^−1^)	C_found_ (µg L^−1^)	RR ^i^ (%)	RSD (%)
Amitriptyline	Nd ^ii^	10.0	9.3	92.7	2.1
		30.0	31.0	103.3	3.7
Nortriptyline	Nd	10.0	9.7	97.0	2.9
		30.0	27.4	91.3	3.6
Imipramine	Nd	10.0	9.5	94.8	4.5
		30.0	30.6	102.1	5.3
Desipramine	Nd	10.0	9.6	96.4	2.7
		30.0	29.2	97.3	1.9
Maprotiline	Nd	10.0	9.9	98.6	4.6
		30.0	31.1	103.6	3.4
Sertraline	32.0	10.0	41.3	93.2	5.7
		30.0	62.7	102.4	4.4

^i^ RR: Relative recovery (RR% = $\frac{C_{\mathrm{found}}-C_{\mathrm{real}}}{C_{\mathrm{added}}}\times 100$); ^ii^ Nd: not detected.

**Table 4:** Obtained results from the determination of TCAs in the urine sample taken 2 h after consumption of imipramine spiked at two different concentration levels.

Analyte	C_real_ (µg L^−1^)	C_add_ (µg L^−1^)	C_found_ (µg L^−1^)	RR (%)	RSD (%)
Amitriptyline	Nd	25.0	24.7	98.8	2.3
		50.0	50.6	101.2	3.9
Nortriptyline	Nd	25.0	24.4	97.6	5.3
		50.0	48.5	97.0	4.4
Imipramine	47.2	25.0	72.7	102.0	2.3
		50.0	97.1	99.8	1.9
Desipramine	Nd	25.0	24.1	96.4	4.6
		50.0	48.9	97.8	5.6
Maprotiline	Nd	25.0	24.3	97.2	4.7
		50.0	48.3	96.6	6.3
Sertraline	Nd	25.0	26.3	105.2	1.1
		50.0	49.8	99.6	2.3

**Table 5:** Obtained results from the determination of TCAs in plasma sample spiked at three different concentration levels.

Analyte	C_real_ (µg L^−1^)	C_add_ (µg L^−1^)	C_found_ (µg L^−1^)	RR (%)	RSD (%)
Amitriptyline	Nd	1.0	1.0	103.1	2.1
		50.0	49.6	99.2	3.6
		250.0	242.5	97.0	2.7
Nortriptyline	Nd	1.0	1.0	96.9	4.2
		50.0	49.1	98.2	4.5
		250.0	242.8	97.1	3.9
Imipramine	Nd	1.0	1.0	98.5	1.3
		50.0	52.1	104.2	2.2
		250.0	249.5	99.8	2.4
Desipramine	Nd	1.0	1.0	96.3	4.2
		50.0	47.7	95.4	3.6
		250.0	241.8	96.7	4.5
Maprotiline	Nd	1.0	0.9	94.6	5.2
		50.0	47.7	95.3	4.4
		250.0	237.3	94.9	3.9
Sertraline	Nd	1.0	1.0	99.9	1.1
		50.0	50.6	101.1	2.6
		250.0	247.3	98.9	1.9

**Table 6:** Obtained results from the determination of TCAs in well water sample spiked at three different concentration levels.

Analyte	C_real_ (µg L^−1^)	C_add_ (µg L^−1^)	C_found_ (µg L^−1^)	RR (%)	RSD (%)
Amitriptyline	Nd	1.0	1.0	96.1	8.1
		50.0	47.1	94.2	3.6
		250.0	258.5	103.4	6.7
Nortriptyline	Nd	1.0	0.9	93.0	4.3
		50.0	50.9	101.7	5.6
		250.0	244.0	97.6	1.9
Imipramine	Nd	1.0	1.0	95.1	2.4
		50.0	48.6	97.2	3.5
		250.0	247.5	99.0	1.3
Desipramine	Nd	1.0	1.0	102.2	1.7
		50.0	49.3	98.5	2.6
		250.0	248.3	99.3	2.2
Maprotiline	Nd	1.0	1.0	97.8	3.8
		50.0	52.7	105.4	4.1
		250.0	235.5	94.2	3.9
Sertraline	Nd	1.0	1.0	97.6	1.1
		50.0	49.6	99.1	2.0
		250.0	254.3	101.7	2.7

## Data Availability

All the relevant data have been included in the manuscript and the supporting material.

## Supplementary Materials

The following supporting information can be downloaded at: https://www.mdpi.com/article/10.3390/bios13010139/s1, including Figure S1: The structure and electrodeposition reaction of PEDOT-GO; Figure S2: Thermogravimetric analysis of PEDOT-GO; Figure S3: Zeta potential analysis of PEDOT-GO; Figure S4: The concentration-time curve of founded imipramine in urine samples taken at different hours after consumption; Table S1: Selected ions and retention times of TCAs employed in SIM mode of GC–MS.

## Abbreviations

Amitriptyline (Ami), attention-deficit/hyperactivity disorder (ADHD), bone marrow aspirate (BMA), conductive polymers (CPs), desipramine (Des), direct immersion SPME (DI-SPME), flame ionization detector (FID), gas chromatography–mass spectrometry (GC–MS), gas chromatograph system (GC), graphene oxide (GO), electromembrane extraction (EME), electromembrane surrounded solid phase microextraction (EM-SPME), imipramine (Imi), lab-on-chip (LOC), limits of detection (LODs), linear dynamic ranges (LDRs), maprotiline (Map), micro-total-analysis system (µ-TAS), nortriptyline (Nor), obsessive–compulsive disorder (OCD), poly(3,4-ethylenedioxythiophene) (PEDOT), poly(3,4-ethylenedioxythiophene)–graphene oxide (PEDOT-GO), post-traumatic stress disorder (PTSD), premenstrual syndrome (PMS), relative recoveries (RRs), relative standard deviations (RSDs), selected ion monitoring mode (SIM), sertraline (Ser), supported liquid membrane (SLM), thermogravimetric analysis (TGA), three-liquid-phase extraction (TLPE), tricyclic antidepressants (TCAs), zeta potential (ZP). Notations Point of zero charge (pH_pzc_), ion balance (χ), negative (−), positive (+).
