# Supplementary material for: On-Chip Electromembrane Surrounded Solid Phase Microextraction for Determination of Tricyclic Antidepressants from Biological Fluids Using Poly(3,4-ethylenedioxythiophene)—Graphene Oxide Nanocomposite as a Fiber Coating
Source: Biosensors (Basel). 2023 Jan 14;13(1):139. doi: 10.3390/bios13010139 (PMC9856149; doi:10.3390/bios13010139)
Supplement: Supplementary file 1 [file biosensors-13-00139-s001.zip › biosensors-2078229-supplementary.pdf]

Supplementary Material

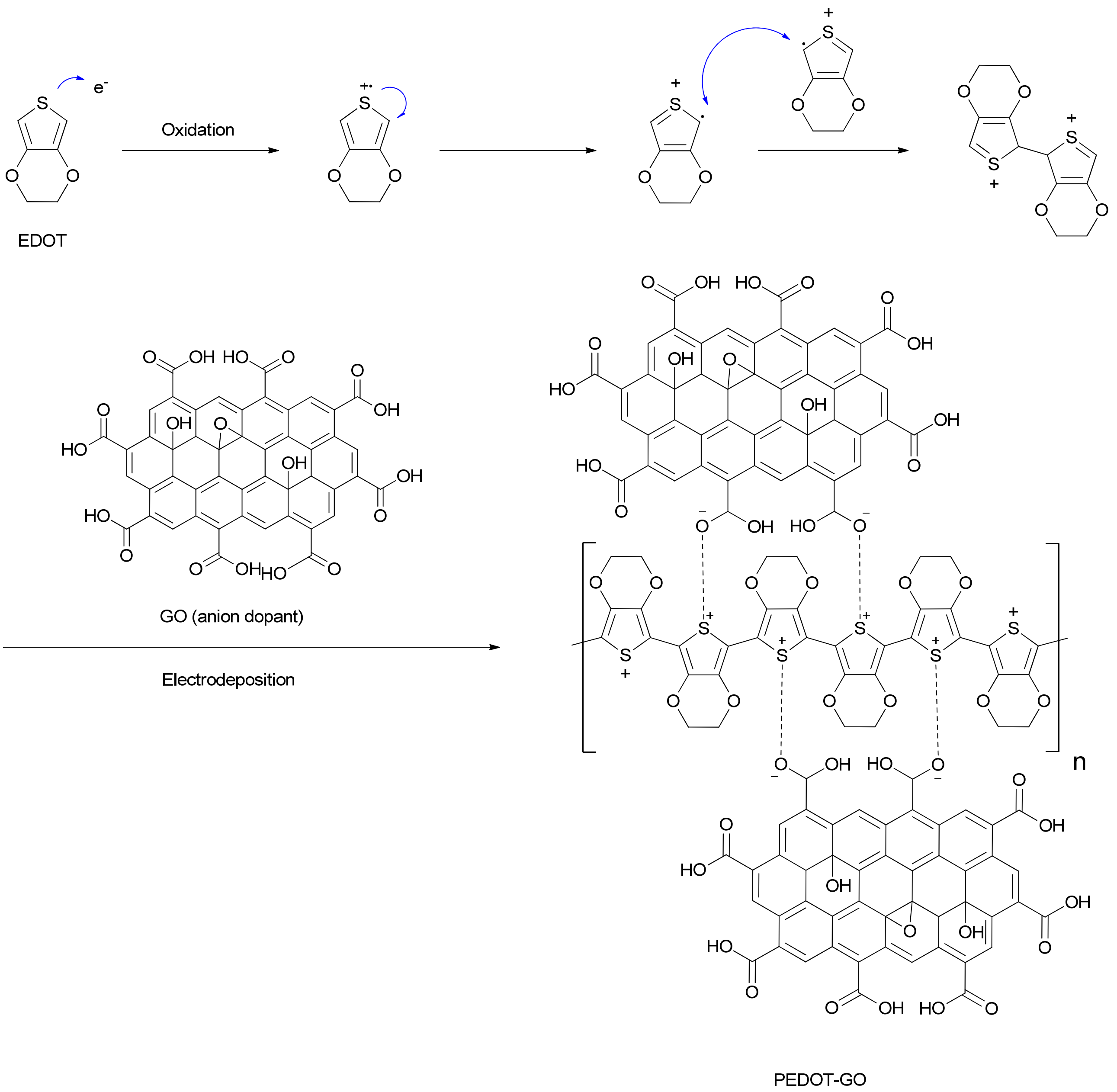

Figure S1. The structure and electrodeposition reaction of PEDOT–GO.

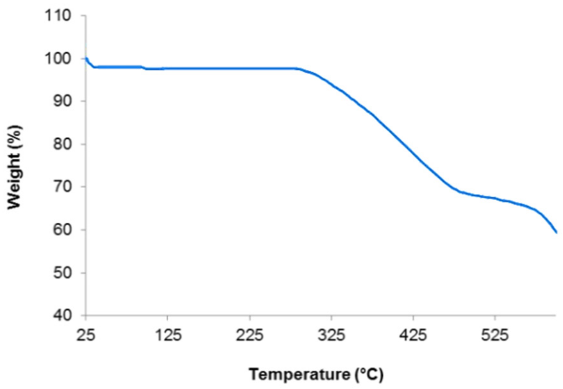

Figure S2. Thermogravimetric analysis of PEDOT–GO.

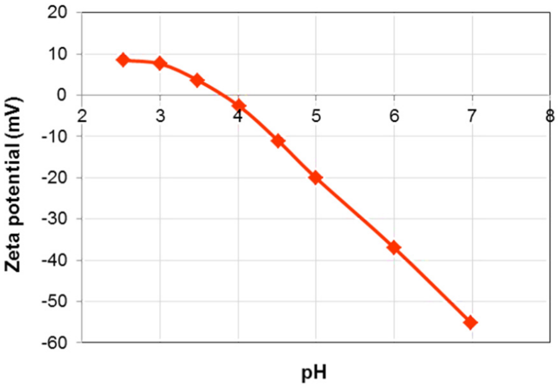

Figure S3. Zeta potential analysis of PEDOT–GO.

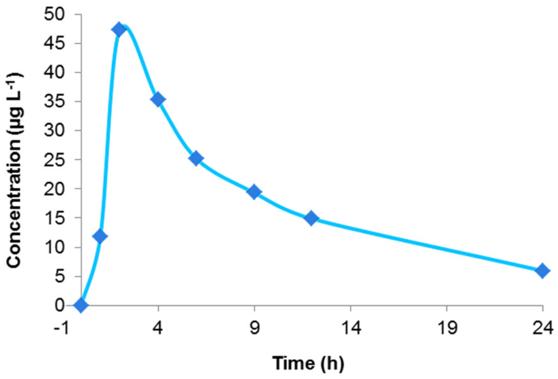

Figure S4. The concentration–time curve of founded imipramine in urine samples taken at different hours after consumption.

Table S1. Selected ions and retention times of TCAs employed in SIM mode of GC–MS.

Analyte	Quantifier (m/z)	Qualifier (m/z)	Retention time (t_R, min)
Amitriptyline	58.0	202.0, 215.0, 189.0	17.3
Nortriptyline	44.1	215.1, 202.1, 189.0	17.4
Imipramine	234.0	85.0, 193.0, 130.0	17.5
Desipramine	195.0	208.0, 234.0, 220.0	17.7
Maprotiline	44.0	203.0, 59.0, 70.0	18.3
Sertraline	274.0	262.0, 159.0, 304.0	18.5
